# Pivotal regulators of tissue homeostasis and cancer: macrophages

**DOI:** 10.1186/s40164-017-0083-4

**Published:** 2017-08-08

**Authors:** Yulei Chen, Xiaobo Zhang

**Affiliations:** College of Life Sciences, Zhejiang University, Hangzhou, 310058 People’s Republic of China

**Keywords:** Macrophage, Phenotype, Polarization, Homeostasis, Cancer

## Abstract

Macrophages are an essential component of innate immunity and play a vital role in inflammation and host defense. Based on immunological responses, the macrophages are classified into “activated” macrophage (M1 macrophages) participating in the responses of type I helper T (Th1) cells to pathogens and “alternatively activated” macrophages (M2 macrophages) in response to interleukin (IL)-4 and IL-13. In this review, we discuss the origin, classification and function of macrophages. We also discuss the mechanisms underlying polarization of different macrophage subtypes, including transcriptional, epigenetic and post-transcriptional regulation.

## Background

Since the discovery of macrophages by Metchnikoff in 1908 and their name conveys the ability to engulf foreign substances, biologists have been occupied with the concept of macrophages as regulators of the innate immune system [1]. To date, it is known that macrophages are not only scavengers of pathogens and dead cells but also important components of the tumor microenvironment, where they regulate tumor progression, matrix remodeling, angiogenesis and metastasis [2]. As the most plastic cells of the haematopoietic system, macrophages are found in all tissues and show great functional diversity. Although macrophages of various tissues are morphologically distinct from one another and have different transcriptional profiles and functional abilities, they are all required for the maintenance of homeostasis [3, 4]. However, the functions of macrophages can be subverted by chronic inflammation, resulting in a causal association of macrophages with disease states. Therefore, revealing the biological roles of macrophages can contribute to understanding the heterogeneity and function of macrophages.

## Origin of macrophages

Macrophages have a broad role in the maintenance of tissue homeostasis, through the clearance of damaged cells and tissues and the repair of tissues. They are active in biosynthesis and express a wide range of receptors, which recognize foreign materials as well as normal and abnormal cells. In fact, macrophages are present in almost all tissues [1]. Macrophages differentiate from circulating peripheral blood mononuclear cells (PBMCs), which migrate into tissue in response to inflammation or in the steady state. These PBMCs develop from a haematopoietic stem cell in the bone marrow that is the precursor of many different cell types, including granulocytes, dendritic cells, macrophages and mast cells. Monocytes develop from haematopoietic stem cells, and then sequentially give rise to granulocyte/macrophage colony-forming units, monoblasts, pro-monocytes and finally monocytes. Afterwards, monocytes are released from the bone marrow into the bloodstream [5, 6]. In the blood, monocytes are not a homogeneous population of cells. Although monocyte heterogeneity is not fully understood, it turns that monocytes migrate from the blood into tissues to differentiate into long-lived tissue-specific macrophages of the bone (osteoclasts), central nervous system (microglial cells), alveoli, gastrointestinal tract, connective tissue (histiocytes), liver (Kupffer cells), peritoneum and spleen [7]. Macrophages differ morphologically and phenotypically in different organs. Through endocytosis, phagocytosis and secretion of various products, including growth factors, cytokines and metabolites, macrophages perform both trophic and toxic functions, thus serving as a widely distributed mononuclear phagocyte during individual development and throughout the whole life.

## Classification of macrophages

Activation of macrophages is a key area of tissue homeostasis, disease pathogenesis and inflammation. Differentiation and activation of macrophages depend on specific growth factors, receptors, signaling pathways and transcription factors. Over the last decades, diverse terms have been applied to macrophage activation, where toll-like receptor (TLR) agonist or cytokine treatment produces distinct patterns of gene and protein expression in macrophages [8]. M1- and M2-polarized macrophages also have distinct features in terms of the metabolism of iron, folate and glucose [9]. Besides, macrophages possess the ability to change their activation states in response to growth factors, cytokines, microbes, microbial products and other modulators [10, 11]. Macrophage activation is involved in the outcome of many diseases, including metabolic diseases, autoimmune diseases, cancers and infections.

Some specific cytokines, including granulocyte macrophage colony stimulating factor (GM-CSF), tumor necrosis factor α (TNF-α) and interferon-γ (IFN-γ) alone or together with lipopolysaccharide (LPS), can activate macrophages into M1 subtype [10]. Classical macrophage activation is characterized by high capacity to present antigen, high interleukin-12 (IL-12) and IL-23 production, low IL-10 production and consequent activation of a polarized type I response [12]. It is also accompanied with high production of inflammation cytokines (IL-1, TNF-α and IL-6) and toxic nitric oxide (NO) and reactive oxygen species (ROS). M1 macrophages mediate the defense of animals against a variety of pathogens and play a key role in anti-tumor immunity [12].

As reported, IL-4 and IL-13 are found to be more than simple inhibitors of macrophage activation, in that they induce a distinct activation program, known as “alternative activation” [13]. Moreover, other cytokines such as IL-33 and IL-25 enhance alternatively activated macrophage induction indirectly through T helper 2 (Th2) cells [13, 14]. Studies have showed that alternatively activated macrophages present M2 phenotype. M2 cells are typically IL-12^low^, IL-23^low^ and IL-10^high^ and generally characterized by low production of pro-inflammatory cytokines (IL-1, TNF-α and IL-6) [12]. M2 macrophages have high levels of scavenger-, mannose-, and galactose-type receptors, and the arginine metabolism is shifted to polyamines and ornithine [15]. In general, M2 macrophages are a component of polarized Th2 responses. Hence, M2 macrophages function in a range of physiologic and pathological processes, including homeostasis, anti-inflammation, repair, metabolic functions and malignancy.

## Underlying mechanisms of macrophage polarization

**Fig. 1:**
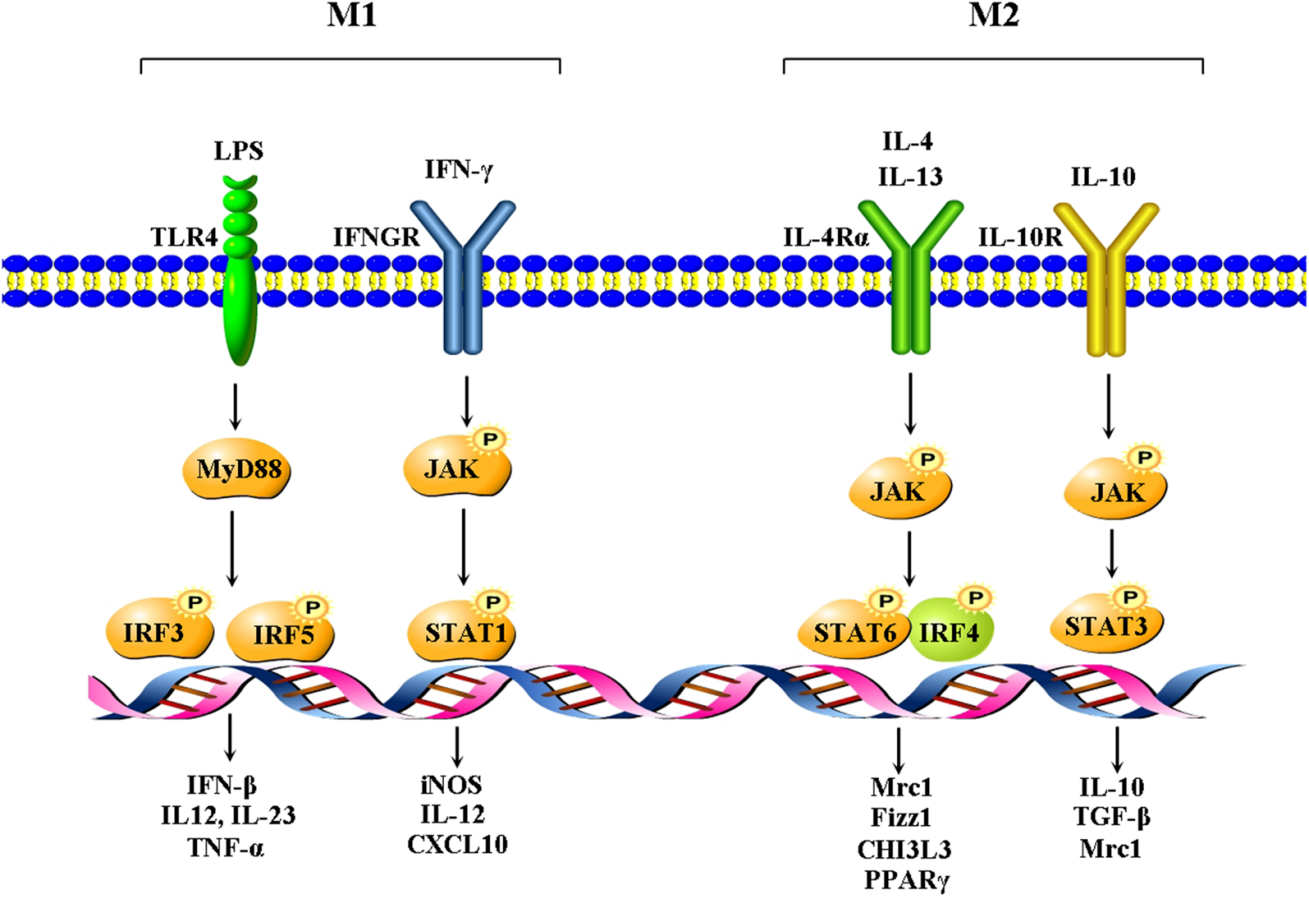
Molecular pathways of macrophage polarization. LPS and IFN-γ trigger the activation of TLR4 and IFN-γ receptor (IFNGR) pathways and induce the phosphorylation of the transcription factors IRF3, IRF5 and STAT1, leading to the transcription of M1-related genes. IL-4 and IL-13 signaling pathway is triggered via IL-4Rα to activate STAT6 and IRF4, thus regulating the expression of M2-related genes. IL-10 signals through IL-10 receptor (IL-10R) to activate STAT3, thereby triggering M2-like macrophage polarization

A network of signaling molecules, transcription factors, epigenetic mechanisms, and post-transcriptional regulators underlies the different phenotypes of macrophages (Fig. 1). As reported, IFN-γ triggers the phosphorylation and dimerization of signal transducer and activator of transcription 1 (STAT1), thus initiating the transcription of M1-associated genes (*iNOS*, *Il12* and *CXCL10*) [16] (Fig. 1). LPS stimulation results in IFN regulatory factor 3 (IRF3) activation via Toll-like receptor adaptor molecule 1 (TICAM1)-dependent Toll-like receptor 4 (TLR4) motivation [17] (Fig. 1). Hence, the IFN-β expression and activation of STAT1 and STAT2 are initiated. Notch-RBP-J signaling also controls expression of the transcription factor IRF8 that induces downstream M1 macrophage-associated genes after TLR4 activation [17]. Besides, M1 macrophages upregulate IRF5, which is essential for induction of cytokines (IL-12, IL-23 and TNF-α) involved in eliciting Th1 and Th17 responses [18]. However, IL-4 and IL-13 treatment leads to the production of M2 macrophages via STAT6 signaling pathway [19]. Activated STAT6 in turn recruits IRF4 and activates transcription of genes typical of M2 macrophages, e.g., mannose receptor C1 (*Mrc1*), resistin-like α (*Fizz1*), chitinase 3–like 3 (*Chi3l3*) and peroxisome proliferator-activated receptor γ (*PPARg*). The interaction between PPARγ and STAT6 facilitates DNA binding and the expressions of various M2-related genes [19] (Fig. 1). IL-6 and leukemia-inhibitory factor, present at high concentrations in ovarian cancer ascites, differentiate monocytes into M2 macrophages by increasing macrophage colony-stimulating factor consumption [20]. IL-10 can activate STAT3-mediated gene expressions, such as *Il10*, *Tgfb1* and *Mrc1*, which are associated with M2 phenotype [12, 21] (Fig. 1).

Macrophage development, polarization and activation are also controlled by epigenetic changes. Epigenetic regulation is typically mediated by post-translational modifications (methylation, acetylation, and phosphorylation) of histones and other chromatin proteins that bind DNA. The methylation and hydroxymethylation of CpG DNA motifs alter gene expression in macrophages [22]. During M1 polarization, master transcription factors, such as PU.1 and CCAAT/enhancer binding protein α (C/EBPα), bind to and open the regulatory regions (promoters and enhancers) of M1-related genes [23]. Enhancers are marked by H3K4me1, PU.1 and open chromatin, as demonstrated by high sensitivity of DNase I digestion [23]. Furthermore, TLR stimulation results in the release of the above epigenetic ‘brakes’, for example concomitant induction or activation of demethylases such as JMJD3, JMJD2d, PHF2 and AOF1 that erase the negative histone marks H3K9me3, H3K27me3 and H4K20me3 [24]. Alternative activation of macrophages is mediated by histone demethylase JMJD3, which facilitates the expression of M2-promoting transcription factor IRF4 by removing negative H3K27me3 marks at the *Irf4* locus [25]. By contrast, HDAC3 acts as a suppressor on IL-4-induced M2 polarization by deacetylation of enhancers of IL-4-induced genes [25]. Therefore, both histone methylation and acetylation are important for M2 polarization.

MicroRNAs (miRNAs) are noncoding small RNAs that play important gene-regulatory roles in animals and plants by pairing to the mRNAs of protein-coding genes to direct their post-transcriptional repression [26]. It is increasingly clear that many miRNAs display tissue- or cell-type-specific expression patterns in cell proliferation, differentiation, and metabolism [27]. Upon LPS and IFN-γ stimulation, the expression levels of miRNAs involved in M1 phenotype polarization are significantly increased, including miR-155, miR-125a/b and let-7e [28–30]. As reported, miR-155 enhances *Tnfa* mRNA stability as inhibition of miR-155 decreases *Tnfa* mRNA half-life, while overexpression of miR-155 increases *Tnfa* mRNA [31]. miR-125a/b directly targets the negative NF-κB regulator TNF alpha-induced protein 3 (TNFAIP3), thus reinforcing NF-κB transcriptional activity [32]. miR-146, miR-9, miR-21 and miR-147 participate in M1 polarization by forming a negative feedback loop with NF-κB [33, 34]. Moreover, miR-223 can be induced by LPS and in turn inhibits the activation of M1 macrophages [35]. On the other hand, miR-187, miR-378-3p and miR-511-3p are engaged in M2 activation [36, 37]. miR-187 recruits *Tnfa* mRNA into RNA induced silence complex (RISC), leading to the degradation of this mRNA. AKT1 signaling suppresses the IL-4-induced M2-realted genes’ expressions, e.g., *Mrc1*, *Fizz1* and *Chi3l3* [38]. miR-378-3p involves in the M2 macrophage activation by targeting AKT1 signaling pathway [37]. miR-511-3p locates in the fifth intron of *Mrc1* gene and is co-transcribed with *Mrc1*, indicating that miR-511-3p is a typical alternatively activated miRNA during macrophage alternative activation [36].

## Macrophages and tissue homeostasis

Mature macrophages are located throughout the body and perform important functions of immune surveillance. They survey their immediate surroundings for signs of tissue damage or invading organisms and are subjected to stimulating immune cells to respond when danger signals are detected by cell surface receptors [39, 40]. Following tissue injury or infection, the macrophages usually exhibit a pro-inflammatory phenotype and secrete pro-inflammatory mediators, such as TNF-α, IL-1, ΝΟ and ROS, which participate in the activation of various antimicrobial mechanisms. Moreover, IL-12 and IL-23 are expressed by activated macrophages and further influence the polarization of Th1 and Th17 cells [41].

In addition to fighting pathogen infections, resident tissue macrophages are involved in maintaining healthy tissues by removing dead and dying cells and toxic substances. Macrophages are selective of the substance that they eliminate by recognition of cell receptors with ligands [42]. During phagocytosis of macrophages, pattern recognition receptors (PRRs) recognize signals of invading pathogens, foreign substances and dead cells. Afterwards, transcriptional mechanisms are activated that lead to phagocytosis and release of cytokines, chemokines and growth factors. Besides, macrophages also secret numerous molecules, including complement and Fc receptors, C3b and antibodies [2]. Therefore, macrophages monitor and respond to changes in their environment by surface receptors and secreted molecules.

## Macrophages in cancers

If the inflammatory macrophage response against infection or injury is not quickly controlled, it will become pathogenic and leads to disease progression. To impede the damage of the inflammatory response, macrophages usually undergo apoptosis or transform into an anti-inflammatory phenotype that facilitates wound healing. It is becoming clear that the mechanisms underlying the conversion of pro-inflammatory and anti-inflammatory macrophages have a major effect on the progression and resolution of chronic diseases, including asthma, atherosclerosis, fibrosis, autoimmune diseases and cancer [43, 44]. Here, this review focuses on the roles of macrophages in cancer initiation and progression.

Cancer is a hyperproliferative disorder that involves morphological cellular transformation, uncontrolled cellular proliferation, angiogenesis and metastasis. In the tumor tissues, except for cancer cells, there are fibroblasts, endothelial cells and immune cells, which constitute tumor microenvironment (TME) [45]. Macrophages are the major immune cells in TME. More and more evidences show that macrophage density of TME correlates with cancer progression and poor prognosis [46–49]. During tissue injury or infection, M1 macrophages produce a series of proinflammatory cytokines such as IL-6, TNF-α, ΝΟ and ROS [50]. On one hand, ΝΟ and ROS are highly toxic for microorganisms as well as adjacent tissues and lead to aberrant inflammation. On the other hand, they can induce a mutagenic environment and genetic instability of adjacent epithelial cells by inhibiting the function of p53 and by increasing the activity of DNA methylase, which leads to an increment on CpG island methylation and an erroneous gene transcription [51]. TNF-α produced by M1 macrophages promotes tumor cell proliferation and neoangiogenetic abilities through the induction of genes encoding anti-apoptotic molecules [52]. M1 macrophages also contribute to constitutive activation of transcription factors, such as NF-κB and STAT3 [53, 54]. NF-κB activation in tumor cells can promotes tumor progression by enhancing their aggressive potential and increase the transcriptions of proinflammatory and angiogenetic genes, for example IL-12, TNF-α and iNOS [53]. Activation of STAT3 leads to resistance to apoptosis in tumor cells and to a tolerant tumor environment [54]. Besides, M1 macrophage-secreted epidermal growth factor (EGF), platelet-derived growth factor (PDGF), hepatocyte growth factor (HGF) and transforming growth factor β (TGF-β) facilitate epithelial growth and survival [55]. Herein, chronic inflammation induces cancer occurrence and M1 macrophage is a vital participant in this process.

Once tumors become established, they cause macrophage differentiation so that the macrophages change from M1 phenotype to M2 phenotype that promotes tumor progression and malignancy. Cyclooxygenase 2 (COX-2) has been shown to be involved in changing the macrophage phenotype from M1 to M2 [56, 57]. M2 cells develop defective NF-κB activation during tumor progression by constitutive formation of p50 homodimers, leading to a reduced expression of iNOS accompanied by an impaired ability to produce NO and a reduced TNF-α secretion [58]. The Notch signaling pathway is also involved in the generation of M2-macrophages [59]. Notch is up-regulated in M1 macrophages, which leads to increased IL-12 production. M2 macrophages in progressing tumors have been shown to down-regulate Notch [59]. Cytokines are involved in the differentiation of M2 macrophages. CXCL12, which is highly produced in the tumor environment, has been shown to not only mediate recruitment and migration of monocytes to the tumor tissue, but also participate in differentiating macrophages towards immunosuppressive M2 macrophages by up-regulating chemokine [C–C motif] ligand 1 (CCL1) expression [60]. M2 macrophages express a variety of proangiogenic cytokines such as vascular endothelial growth factor (VEGF), basic fibroblast growth factor (bFGF), IL-8 and enzymes, including matrix metalloproteinases (MMPs) and COX-2 [61]. VEGF deficiency in macrophages significantly inhibits tumor angiogenesis, while overexpression of VEGF facilitates tumor angiogenesis and progression [62]. Besides, macrophages also participate in the junction and remodeling of new blood vessels [63].

Metastasis, which represents the migration of cancer cells from the primary tumor to a distant organ or tissue, is the most frequent cause of poor prognosis and death for patients with cancer. The steps of metastasis include tumor cell adhesion and invasion of basement membranes and the surrounding tissue, intravasation into blood vessels, survival in the bloodstream, extravasation from blood vessels, and growth at different organ sites [64]. Cancer researchers have focused their studies on macrophage- secreted factors that potentially influence cancer cell migration, invasion or adhesion. To date, EGF, CCL18, IL-18, IL-1β and IL-8 secreted by tumor-associated macrophages (TAMs) have been extensively investigated [65–68]. Our previous study indicated that M2 macrophages-secreted CHI3L1 (chitinase 3-like protein 1) protein specifically bound to the interleukin (IL)-13 receptor α2 chain (IL-13Rα2) of gastric and breast cancer cells, thus promoting cancer metastasis [69]. Paracrine loops of EGF/colony-stimulating factor 1 (CSF-1) and CCL18/granulocyte-macrophage colony-stimulating factor (GM-CSF) between M2 macrophages and cancer cells have been shown to increase carcinoma cell invasion [70]. Besides, macrophage-produced osteonectin in the tumor extracellular matrix (ECM) is engaged in cancer cell migration by facilitating cancer cell adhesion to fibronectin [71].

## Relationship between myeloid-derived suppressor cells (MDSCs) and TAMs in cancer

As discussed above, cancer initiation and progression are assisted by TAMs. A high frequency of TAMs is associated with poor prognosis in many tumors [72, 73]. MDSCs have attracted increased attention, and their presence in the blood of cancer patients is emerging as a simple prognostic marker to monitor clinical outcome and therapy. MDSCs are characterized by myeloid origin, heterogeneity and ability to downregulate immune responses in cancer [74]. Immunosuppressive MDSCs with monocytic features can traffic from bone marrow to tumor tissues, mainly through the same chemokine pathway of CCR2/CCL2 axis with TAMs [75].

In tumor-bearing hosts, generation of MDSC and TAM requires the integration of two types of signals: factors that expand myeloid precursors and factors that activate immune-regulatory programs [4, 76–78]. CSF1, granulocyte-CSF (G-CSF), and GM-CSF are the three major regulators of myeloid lineage proliferation and differentiation [76]. G-CSF promotes the differentiation of myeloid precursors into MDSCs. The master factor for TAM recruitment and programming in the TME is CSF1 [4]. IL-4 and IL-13 participate in both TAM and MDSC survival and the acquisition of an immunosuppressive phenotype [77]. Metabolic environmental signals can also modulate the intratumoral distribution of myeloid cells [78].

The activities of MDSC and TAM not only contribute to an immunosuppressive environment that keeps T cells at bay and protect tumors from the effects of the immune system, but include mechanisms that sustain and promote tumor growth and metastasis [79]. TAMs and MDSCs exert their immunosuppressive effects in an antigen-specific or antigen-nonspecific manner [79]. To sustain the immunosuppressive environment, TAMs and MDSCs secrete kinds of chemokines acting on CCR5 and CCR6, which are involved in Treg recruitment. MDSCs can also skew macrophages toward an M2 phenotype through a cell contact–dependent mechanism, characterized by decreased production of IL-12 [80]. The downregulation of IL-12 is further enhanced by the macrophages themselves, because TAMs stimulate an additional IL-10 release by MDSCs, thereby creating a negative loop. Therefore, TAMs and MDSCs regulate the intratumoral IL-10 and IL-12 balance, which is critical for triggering T cell responses [81].

## Conclusions

As essential regulators of inflammation and host defense, macrophages exert a dual influence on tumor growth and progression. Plasticity and heterogeneity are hallmarks of the macrophage lineage. Hence, the specific markers used to distinguish M1 and M2 phenotypes require further standardization and development. Previous studies have revealed that adaptive immunity can orchestrate cancer-promoting inflammation and TAMs through various molecular pathways [82]. Thus, the development of effective strategies to block cancer-promoting inflammation or to activate protective innate immunity will contribute to the control of cancer initiation and progression. As cancer is a worldwide problem, screening of specific agents or inhibitors targeting recruitment or cancer-promoting activity of TAMs and MDSCs in TME will be helpful to control cancers [73, 83, 84].

## Abbreviations

Th1 cells: type I helper T cells
IL-4: interleukin-4
PBMCs: peripheral blood mononuclear cells
TLR: toll-like receptor
GM-CSF: granulocyte macrophage colony stimulating factor
TNF-α: tumor necrosis factor α
IFN-γ: interferon-γ
LPS: lipopolysaccharide
NO: nitric oxide
ROS: reactive oxygen species
STAT1: signal transducer and activator of transcription 1
TICAM-1: toll-like receptor adaptor molecule 1
TLR4: toll-like receptor 4
Mrc1: mannose receptor C1
Fizz1: resistin-like α
Chi3l3: chitinase 3–like 3
PPARγ: peroxisome proliferator-activated receptor γ
C/EBPα: CCAAT/enhancer binding protein α
miRNA: microRNA
TNFAIP3: TNF alpha-induced protein 3
PRRs: pattern recognition receptors
TME: tumor microenvironment
EGF: epidermal growth factor
PDGF: platelet-derived growth factor
HGF: hepatocyte growth factor
TGF-β: transforming growth factor β
VEGF: vascular endothelial growth factor
bFGF: basic fibroblast growth factor
COX-2: cyclooxygenase 2
MMPs: matrix metalloproteinases
CCL18: chemokine [C–C motif] ligand 18
TAMs: tumor-associated macrophages
CHI3L1: chitinase 3-like protein 1
IL-13Rα2: interleukin-13 receptor α2 chain
CSF-1: colony-stimulating factor 1
GM-CSF: granulocyte–macrophage colony-stimulating factor
ECM: extracellular matrix
